# Monitoring Breathing and Heart Rate Using Episodic Broadcast Data Transmission

**DOI:** 10.3390/s22166019

**Published:** 2022-08-12

**Authors:** Paweł Janik, Małgorzata A. Janik, Michał Pielka

**Affiliations:** Faculty of Science and Technology, Institute of Biomedical Engineering, University of Silesia in Katowice, ul. Będzińska 39, 41-200 Sosnowiec, Poland

**Keywords:** episodic broadcast transmission, Bluetooth low energy, IoT, breath sensor, heart rate monitoring, transmission control algorithm

## Abstract

The paper presents a wearable sensor for breath and pulse monitoring using an inertial sensor and episodic broadcast radio transmission. The data transmission control algorithm applied allows for the transmission of additional information using the standard PDU format and, at the same time, goes beyond the Bluetooth teletransmission standard (BLE). The episodic broadcast transmission makes it possible to receive information from sensors without the need to create a dedicated radio link or a defined network structure. The radio transmission controlled by the occurrence of a specific event in the monitored signal is combined with the reference wire transmission. The signals from two different types of sensors and the simulated ECG signal are used to control the BLE transmission. The presented results of laboratory tests indicate the effectiveness of episodic data transmission in the BLE standard. The conducted analysis showed that the mean difference in pulse detection using the episodic transmission compared to the wire transmission is 0.038 s, which is about 4% of the mean duration of a single cycle, assuming that the average adult human pulse is 60 BPM.

## 1. Introduction

The development of so-called high technologies influence the development of digital societies. In turn, the digitization of society manifests itself, inter alia, in the increased use of software, mobile devices, personal computers, or network services. Social demand for modern technologies also applies to the areas of pro-health and e-care. Here, digitization absorbs, for example, sensor technologies designed for IoT (Internet of Things) applications [1,2,3]. Sensors of this type enable data transmission to other devices, especially mobile ones. Wearable sensors [4,5,6] help monitor the health of patients from different age groups. Wearable sensors can also support the treatment of chronic diseases, which constitute a serious social problem [7,8,9]. Wearable solutions are also used to monitor vital functions or locomotor activities, which are periodic in nature. For this purpose, for example, inertial sensors (IMU—Inertial Measurement Unit) are used [10,11,12,13]. The process of monitoring the state of the body with the use of wearable solutions involves recording and transmission of measurement data. There are many devices on the market that enable us to save data to the built-in memory, e.g., ECG Holter systems. Such a measurement structure with local data recording is justified by the need to save energy during the long-term operation of battery-powered devices. Nowadays, however, wearable solutions that use radio interfaces are developing dynamically. In this aspect, radio interfaces operating in the ISM bands, which are intended, among others, for industrial, scientific, and medical applications, are of great importance [14]. Taking into account the possibility of cooperation of wearable solutions with, for example, mobile devices or stationary receivers, the 2.4 GHz band is often used. Within this band, many teletransmission standards have been developed, e.g., Wi-Fi, Bluetooth, ZigBee. Energy-saving 2.4 GHz transceivers [15] are also used to transmit data from the patient monitoring process. Teletransmission technologies for the 2.4 GHz band are also used in closed/confined structures of PANs (Personal Area Networks) for sensors with biomedical data transmission [16]. The technological development of wearable systems for monitoring body parameters concerns not only the area of sensor technology or teletransmission standards, but also solutions in the field of flexible wearable antennas [17,18]. In turn, among the wearables with radio data transmission in the 2.4 GHz band, an important role is played by the Bluetooth standard, which is characterized by low energy consumption [19]. In this category, the proposed solutions include, inter alia, structures of wearable sensors with transmission via Bluetooth to smartphones, which then upload data to the server [20]. Research is also carried out with the use of measurement systems, in which Bluetooth transmission is an alternative to Wi-Fi transmission, owing to the use of the Wi-Fi and Bluetooth combo chip ESP32 [21]. There are also wearable solutions in the form of complex sensor systems, in which the Bluetooth standard is implemented as the basic telecommunication standard. Examples include systems monitoring the activity and vital signs during sleep [22] and the BLE Beacons wearable systems which send notifications when Alzheimer’s Disease patients leave a building or room [23]. The miniaturization of electronic systems for radio communication allowed for the dynamic development of wearable solutions, which is additionally stimulated by market demand. Therefore, it is important to develop new teletransmission technologies and procedures to monitor the patient’s body condition. The paper presents a technology that uses a reduced telecommunication architecture based on the energy-saving Bluetooth standard and the related episodic broadcast teletransmission method. Previous papers [24,25] presented the use of connectionless BLE broadcasting to monitor respiratory function. The research was related, inter alia, to reducing the energy consumption of radio transmission. BLE standard transmitters have built-in mechanisms for effective energy management, thanks to the use of impulse transmission. The PDU transmission is performed in a discrete manner and is associated with impulse energy consumption. PDUs are transmitted with declared fixed-value transmission intervals. The paper [24] presents the results of experiments with the use of a BLE transmitter transmitting a PDU with data from respiratory signals. The transmitter is configured for transmission with a fixed transmission interval of 200 ms, which allowed for continuous monitoring of respiratory functions for 6 days using a power supply from a small 3.7 V, 250 mAh battery. This paper also verified the possibility of simultaneous use of several BLE transmitters in the connectionless broadcast mode for monitoring respiratory signals. In turn, the paper [25] verified the impact of the BLE transmission interval in broadcast mode on its energy efficiency in terms of respiratory function monitoring. In the conducted research, the transmission interval of the BLE transmitter was defined successively for 11 values, which in practice allowed for the transmission of 1 PDU per 10 s to 10 PDUs per second. The obtained results showed that, as expected, increasing the transmission interval reduces the energy demand of the BLE transmitter. However, it also has an impact on the quality of mapping the monitored signals. Too large transmission intervals reduce the transfer of data related to the monitored processes. The results of the tests presented in the papers [24,25] showed the usefulness of broadcast transmission in sensor systems for monitoring respiratory functions. At the same time, they raised the question of whether it is possible to send more information without increasing the energy demand for radio data transmission. This issue became the motivation to carry out the experiments and elaborate on their results presented in this article. The new concept is to control the PDU transmission in conjunction with the occurrence of a specific episode as opposed to the standard predefining of a fixed value of the PDU transmission interval. This BLE transmitter control procedure allows for effective management of energy spent on transmission and, at the same time, contains additional information about the monitored processes, which are not saved in the form of data loaded into the PDU.

## 2. Materials and Methods

The structure of measurement systems presented in the article consists of a hardware base and a software layer. First, the variants of the hardware base of the measurement systems used were described (in Section 2.1), taking into account the configuration of sensors (Section 2.2). Then, in Section 2.3, the appropriate logical structures of the software concerning the algorithms implemented both in the sensors and measuring stations, taking into account BLE transmission, were presented.

### 2.1. Measurement Equipment

Three measuring stations were designed for testing the episodic broadcast transmission in the BLE standard. The efficiency of episodic data transmission is presented both with the use of a hardware base implemented in two measuring stations and with the use of ECG signals and respiratory activities generated in the third measuring station. Maxima, conventionally referred to as pulse, were determined in the processed signals.

**Figure 1:**
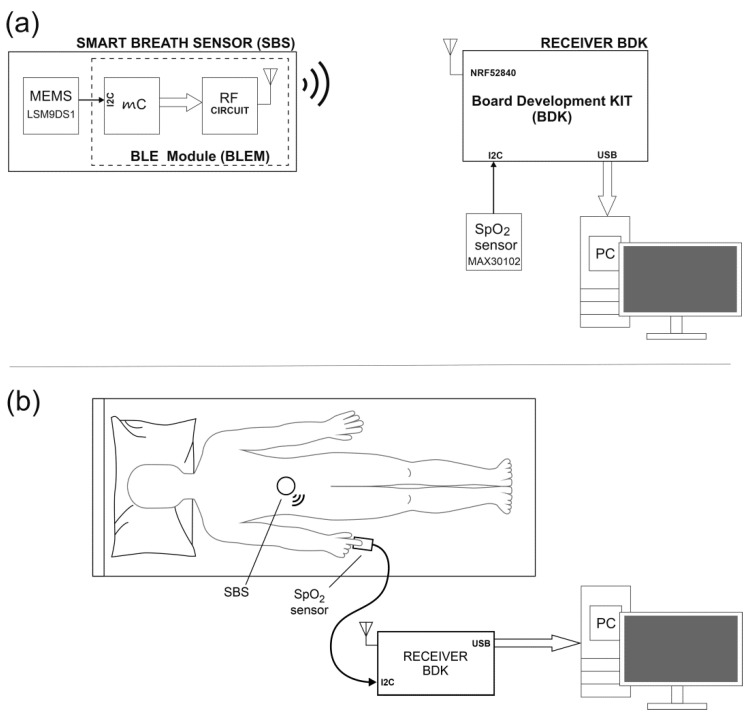
(**a**) Block diagram of the stand for testing the inertial sensor with episodic BLE broadcast transmission with the reference SpO_2_ sensor, (**b**) Visualization of the measuring station and placement of sensors during the experiment.

Figure 1a shows the block diagram of the first station. The main element of the station is a radio sensor (Smart Breath Sensor—SBS) with a reduced architecture [24,25,26,27], that means that only a microcontroller (µC) built into the SoC system (System–on–a–Chip) of the BLE radio module was used. The structure of the radio sensor uses a module based on the nRF52832 system [28]. An inertial sensor (IMU—Inertial Measurement Unit) LSM9DS1 was attached to the microcontroller (µC) of the nR52832 system, which in the measuring system was used for pulse detection. The LSM9DS1 sensor [29] is connected to the µC via the I2C bus. It is an integrated circuit (MEMS-microelectromechanical system) which includes a 3D accelerometer, 3D gyroscope, and 3D magnetometer. LSM9DS1 has a built-in hardware temperature drift correction mechanism for the accelerometer. The data from the three-axis accelerometer, which are transmitted via the RF Circuit, were used for the tests. The station also includes MAX30102 [30], which acts as a reference sensor. It is an SpO_2_ sensor that is connected via the I2C bus to the board development kit NRF52840-DK (BDK) [31]. The applied type of reference sensor (High-Sensitivity Pulse Oximeter and Heart-Rate Sensor) is designed for wearable applications, which was the selection criterion. Additionally, in the MAX30102 sensor, the manufacturer has implemented the ability to control the power supply with diodes, which allows for temperature change compensation. The SpO_2_ sensor requires a dual voltage supply. 3.3 V was taken from the BDK from the VDD NRF pin, whereas a 1.8 V stabilizer was implemented in the sensor PCB circuit. NRF5 SDK and Keil uVision 5 were used to program nRF52832 and NRF52840-DK. The BDK module contains the SoC nRF52840 system, which allows for the reception and integration of data transmitted from the sensors by radio (BLEM) and by wire (MAX30102). BDK was connected via the USB port to a PC on which the data was recorded. Figure 1b shows graphically the method of connecting the sensors to the BDK module and their placement on the body.

**Figure 2:**
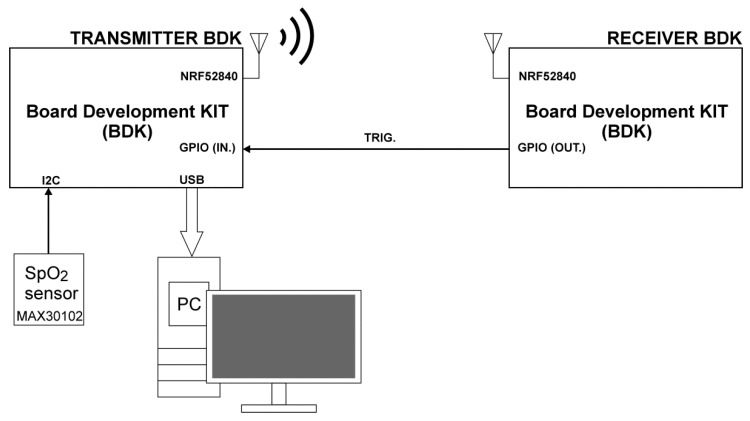
Block diagram of the stand for testing episodic transmission controlled by the signal from the SpO_2_ sensor.

In turn, Figure 2 shows the block diagram of the second measuring station. Only a single MAX30102 sensor was implemented in its structure. This sensor was connected via the I2C bus to the board development kit NRF52840-Preview DK, whose radio module was configured for the connectionless broadcast mode. The BDK transmitter transmits data by radio from the SpO_2_ sensor to the BDK Receiver and by wire—via a USB port to the PC. After receiving the data, the BDK receiver generates the Trig signal, which confirms the reception of a PDU (Protocol Data Unit). The Trig signal is then fed via wire to one of the GPIO inputs of the BDK Transmitter, and the confirmation of data reception is transmitted to the PC via the USB bus. 

**Figure 3:**
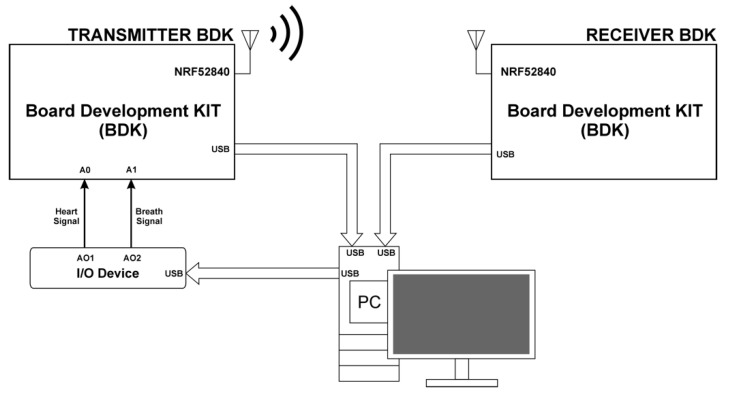
Block diagram of the stand for testing episodic transmission controlled by the digitally generated ECG signal.

The third measuring station presented in Figure 3 was designed to present an exemplary scenario of using episodic BLE broadcast transmission to monitor breathing and pulse. No sensors were implemented in this station, but ECG signals (Heart Signal—HS) and respiratory functions (Breath Signal—BS) were generated. HS and BS signals were generated via the I/O Device—NI USB-6361. The LabView environment was used to control the I/O Device. The HS and BS signals were generated on analogue outputs AO1 and AO2 of the I/O Device. In turn, the analogue outputs AO1 and AO2 were connected to the analogue inputs marked as A0 and A1 of the BDK Transmitter module. Both the Transmitter BDK and Receiver BDK were connected to the PC via the USB interface. As a result, it was possible to monitor sending and receiving of individual PDUs, as well as reconstruct the sent respiratory and pulse signals. The HS signal was generated at a sample rate of 1000 Sa/s, whereas the slow-varying BS signal was generated at a sample rate of 10 Sa/s. The HS digital data were recorded using an ECG simulator—He Instruments TechPatient Cardio V4 with BPM (beats per minute)—60 and BPM Deviations—8%. In turn, the digital data for the BS signal came from the previously carried out measurements (presented in the earlier paper [24]).

### 2.2. Measurement Sensors

**Figure 4:**
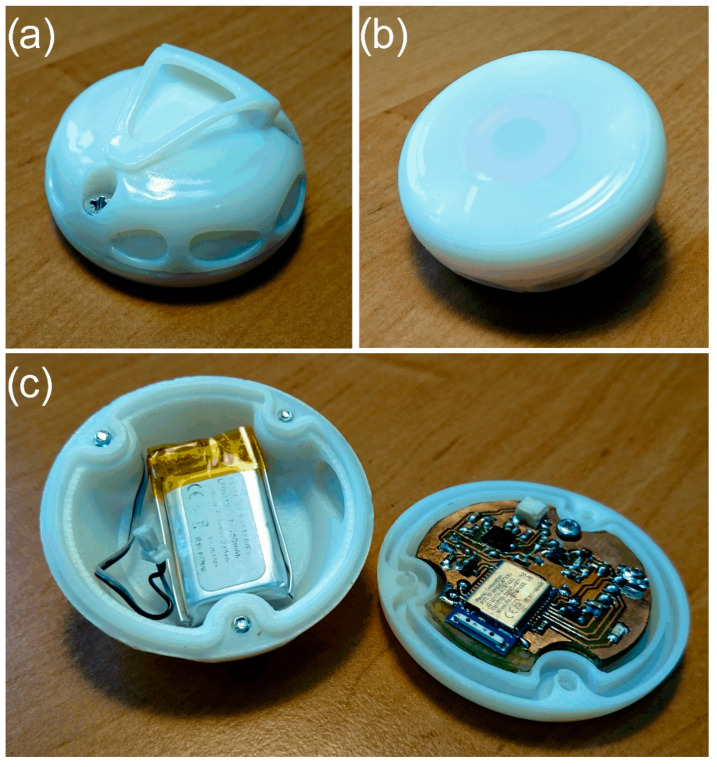
View of the SBS for pulse monitoring with the use of broadcast episodic transmission (**a**) housing—top cover with a handle, (**b**) housing—body, (**c**) sensor circuits in the housing.

The research required the development of printed circuits for commercial sensors (LSM9DS1 and MAX30102). Then, housings were made for the designed electronics using 3D printing. The radio sensor built on the basis of LSM9DS1 enables us to detect the pulse, number of breathing cycles, and body position, especially during sleep. Body movements are limited while resting, which reduces their impact on the vibrations of the anterior abdominal wall on which the IMU sensor was placed. In the part of the paper concerning the first (Figure 1) and the second measuring system (Figure 2), the focus was solely on pulse detection. The breathing cycles and the change of body position were not analysed. Figure 4 shows the structure of the developed radio sensor. The sensor electronics were placed in a two-part housing, one part of which is the battery compartment and has a handle that allows the sensor to be attached, e.g., to clothing (Figure 4a), whereas the second part is the basis for the PCB circuit and provides contact with the human body (with the front abdominal wall) Figure 4b. The electronics are powered by a Li-Po 1s battery (3.7 V, 250 mAh)—Figure 4c. The radio sensor was developed especially for sleep monitoring in children. 

**Figure 5:**
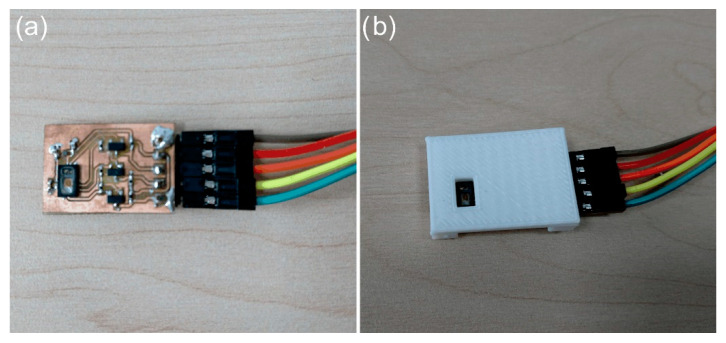
Structure of the SpO_2_ sensor built for testing (**a**) PCB circuit of the sensor, (**b**) flat cover with a measuring slot for the SpO_2_ sensor.

The construction of the sensor PCB circuit designed for the SpO_2_ sensor is shown in Figure 5a. The MAX30102 system was placed on a 20 × 30 mm board, for which a flat overlay with a measuring gap was designed (Figure 5b). The overlay is the distance between the optoelectronic system and the fingertip. The developed PCB module is connected to the NRF52840-Preview DK board (BDK), as shown in Figure 1 and Figure 2.

### 2.3. Sensor Software Logic Diagrams

The episodic broadcast data transmission in the BLE standard is presented using two sample sensors for which embedded software was developed. It should be noted, however, that the use of this type of transmission is not limited to some selected sensors. In turn, the embedded software is not of key importance for the presented content, therefore it was not optimized to obtain the highest measurement accuracy. The embedded software was designed to highlight the BLE episodic broadcast transmission.

**Figure 6:**
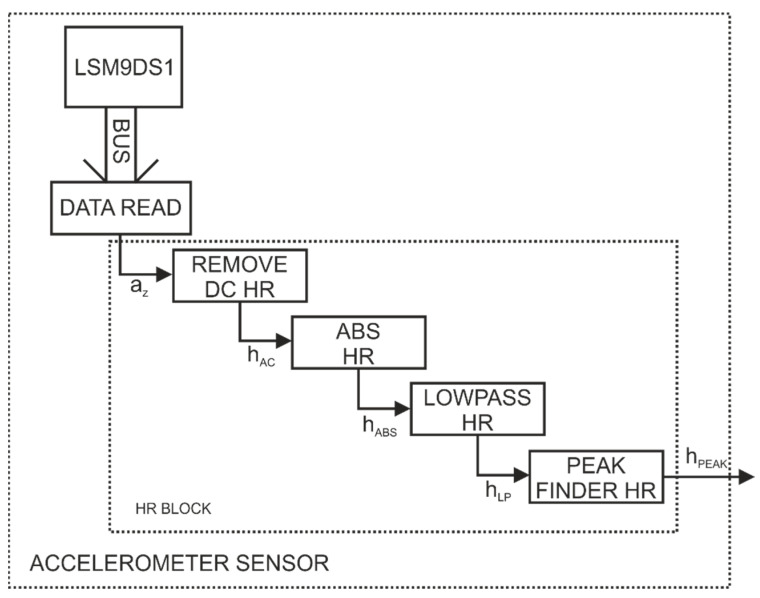
Software logic diagram for the LSM9DS1 sensor.

Figure 6 shows the logic diagram of the software for the LSM9DS1 sensor. The sensor in the implemented system enables us to monitor the pulse, with the LSM9DS1 system accelerometer being used. Pulse detection was performed by placing the sensor (Figure 4) on the front abdominal wall that performs both slow oscillations related to breathing (not analysed in this paper) and fast oscillations related to heart beating (cycle of about 1 s), which were used to control the presented episodic transmission.

Front abdominal wall vibration sampling is 50 Hz. The data from LSM9DS1 are sent to the µC (built in the RF module with an nRF52832 chip) via the I2C bus (BUS). The data processing algorithm (ACCELEROMETER SENSOR) was implemented in the microcontroller, which allows for pulse detection. In order to simplify and speed up the algorithm, the reading of data from the sensor (DATA READ) is performed only from the axis z (a_z_), and then the data are transferred to the HR BLOCK.

In the HR BLOCK, the DC component of the signal is removed first (REMOVE DC HR). For the resulting signal h_AC_, the modulus is calculated from the value (ABS HR), which provides the h_ABS_ signal. This process is followed by signal filtering with a 3 Hz low-pass filter (LOWPASS HR), which yields the h_LP_ signal. The configured low-pass filter enables us to monitor up to 180 heart rate cycles per minute, which was a sufficient value during the conducted research. The last step in the HR block is to determine the local maxima (PEAK FINDER HR) from the h_LP_ signal that represents the pulse. The resulting signal of the HR BLOCK is a sequence of time values h_peak_ at which the pulse peak was detected.

**Figure 7:**
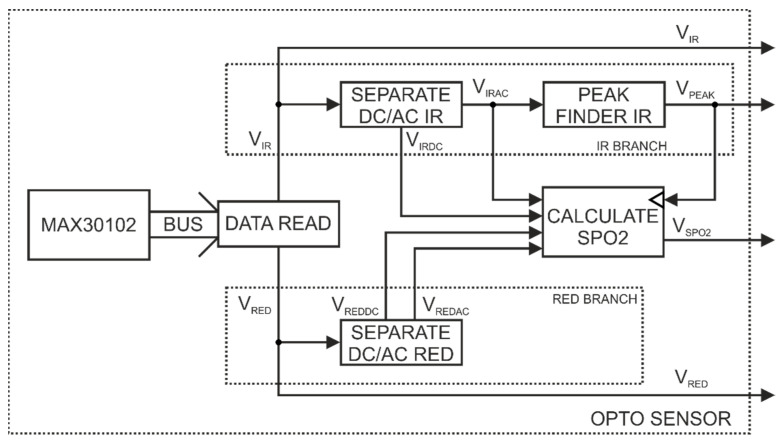
Software logic diagram for the SpO_2_ optoelectronic sensor—MAX30102.

Figure 7 shows the logic diagram of the software developed for the optoelectronic sensor SpO_2_—MAX30102. The sensor was connected to the µC of the SoC—NRF52832 system via the I2C bus (BUS). An algorithm (OPTO SENSOR) was implemented in the microcontroller, which enables us to determine the pulse and blood oxygenation values. The MAX30102 system samples the measurement signal at a frequency of 400 Hz, but averages the values of 8 measurement points, so the actual signal sampling frequency is 50 Hz. The MAX30102 system has a buffer that is read after 24 measurements. The data from the sensor are read (DATA READ) and then processed by the microcontroller. The algorithm processing data from the MAX30102 sensor are divided into two branches. The first one (IR BRANCH) processes the data (*V_IR_*) obtained from the measurements made with the use of an infrared diode, whereas the other one (RED BRANCH) processes the data (*V_RED_*) obtained from the measurements made with the use of a red diode. The *V_IR_* data are used to determine both the pulse and blood oxygenation, whereas the *V_RED_* data are only used for the calculation of the oxygenation value. In the IR BRANCH in the SEPARATE DC/AC IR block, the following components are separated: constant (*V_IRDC_*) and variable (*V_IRAC_*). The next step involves determining the local maxima (*V_PEAK_*) in the block (PEAK FINDER IR) from the *V_IRAC_* signal. The determined local maxima reflect the pulse. The output data from the IR BRANCH are time values at which local maxima were detected (*V_PEAK_*), the constant component (*V_IRDC_*), and the variable component (*V_IRAC_*) of the signal obtained from the measurements made with infrared light.

In turn, in the RED BRANCH in the SEPARATE DC/AC RED block, the following components are separated: the constant (*V_REDDC_*) and the variable (*V_REDAC_*) of the *V_RED_* signal. When a peak (pulse) is detected, the blood oxygenation value is calculated in the CALCULATE SPO_2_ block. This value is calculated on the basis of the constant and variable components of the signals obtained from measurements performed with infrared and red light. Blood oxygenation is calculated according to the Formula (1) recommended by the manufacturer of the MAX30102 system [32].

(1) $$V_{SPO2}=104-17\cdot \left(\frac{\left(\frac{V_{REDAC}}{V_{REDDC}}\right)}{\left(\frac{V_{IRAC}}{V_{IRDC}}\right)}\right)$$

The resulting signal of the OPTO SENSOR algorithm is a sequence of pulse detection times (*V_PEAK_*) and a sequence of values representing blood oxygenation (*V*_*SPO*2_), which were calculated at the times of pulse detection. In Figure 7, the raw data read from the MAX30102 sensor (*V_IR_* and *V_RED_*) are also marked as an output signal.

### 2.4. Logic Diagrams of Measuring Stations 

This section presents the logic diagrams of measuring stations that use the radio sensors with the built-in algorithms described in the previous section. 

**Figure 8:**
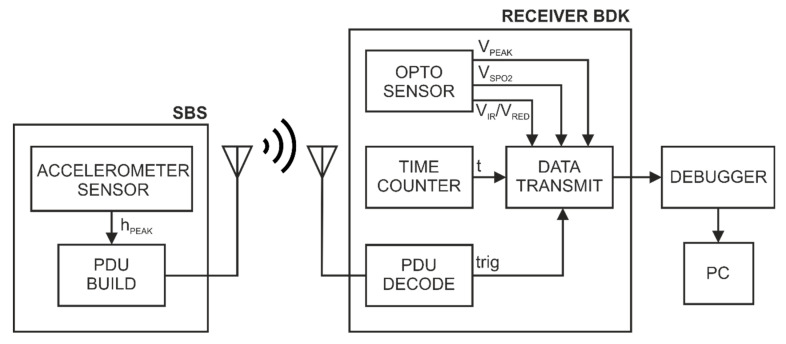
Logic diagram of the stand from Figure 1—for testing the inertial sensor with episodic BLE broadcast transmission.

Figure 8 shows the logic diagram of the station in Figure 1. The logic structure of SBS and the structure of Receiver BDK are separated in the diagram. The SBS enables to monitor breathing and heart rate via the MEMS system—LSM9DS1. The data from the LSM9DS1 system are processed using the ACCELEROMETER SENSOR algorithm, which in turn generates output data to control the radio transmission. The paper presents an example of radio transmission control in broadcast mode by means of pulse detection. In the case of the station in Figure 1, the transmission is controlled by means of the h_PEAK_ signal, which is defined in the ACCELEROMETER SENSOR logic diagram (Figure 6). At the time of pulse detection by the SBS, a data packet (PDU BUILD) is created and sent. The data packet is then received and decoded (PDU DECODE) by a reference circuit—Receiver BDK. The reference system includes the MAX30102 sensor and the previously implemented and described measurement algorithm for this sensor (OPTO SENSOR). The output data of the OPTO SENSOR algorithm are a sequence of time values corresponding to the times of pulse detection (*V_PEAK_*) and the blood oxygenation value (*V_IR_* and *V_RED_*). The reference system includes a time measurement block (TIME COUNTER) that counts the time since the device was started (t). The TIME COUNTER block generates a common time base for measurements performed by the OPTO SENSOR algorithm and moments (trig) representing the receipt of data packets. In the DATA TRANSMIT block, the measurement data are aggregated and then transferred by a debugger (DEBUGGER) to the PC.

**Figure 9:**
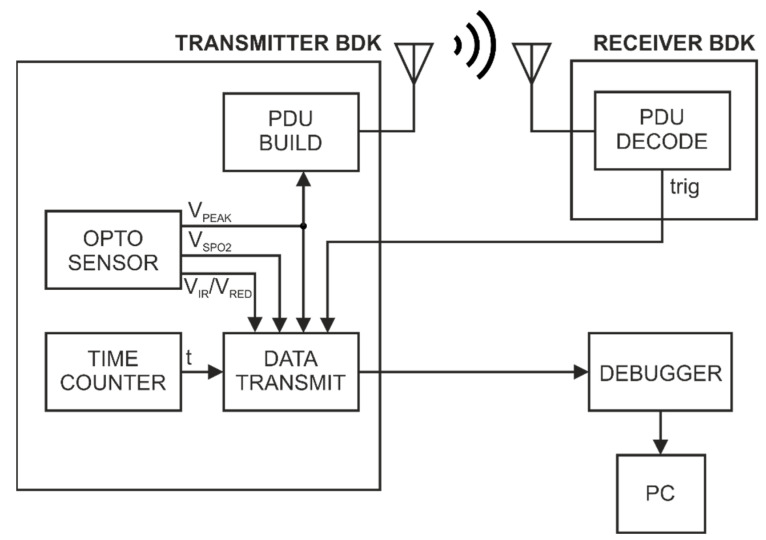
Logic diagram of the stand from Figure 2—for testing broadcast episodic transmission controlled by the signal from the SpO_2_ sensor.

Figure 9 shows the logic diagram of the station in Figure 2. The station uses two BDK modules, on which different software has been implemented—Transmitter BDK and Receiver BDK. The BDK transmitter collects and processes data from the MAX30102 sensor using the OPTO SENSOR algorithm. The output data of the algorithm are a sequence of time values corresponding to the times of pulse detection (*V_PEAK_*), blood oxygenation (*V_SPO_*_2_), as well as raw data obtained from the MAX30102 sensor (*V_IR_* and *V_RED_*). The pulse signal (*V_PEAK_*) is used to control the radio transmission. At the time of pulse detection, a data packet (PDU BUILD) is created and sent. The packet is then received and decoded (PDU DECODE) by the BDK Receiver. In turn, the Transmitter BDK and Receiver BDK are wired via a general-purpose input/output (GPIO). At the time of data packet reception, the Receiver BDK generates an impulse (trig). The BDK transmitter has a time measuring system (TIME COUNTER) that counts the time from starting the device (t). The measurement data from the SpO_2_ sensor and reception moments of data packet are aggregated in the DATA TRANSMIT block. Then the data are sent to the PC via the debugger (DEBUGGER).

### 2.5. Description of Episodic Radio Transmission

**Figure 10:**
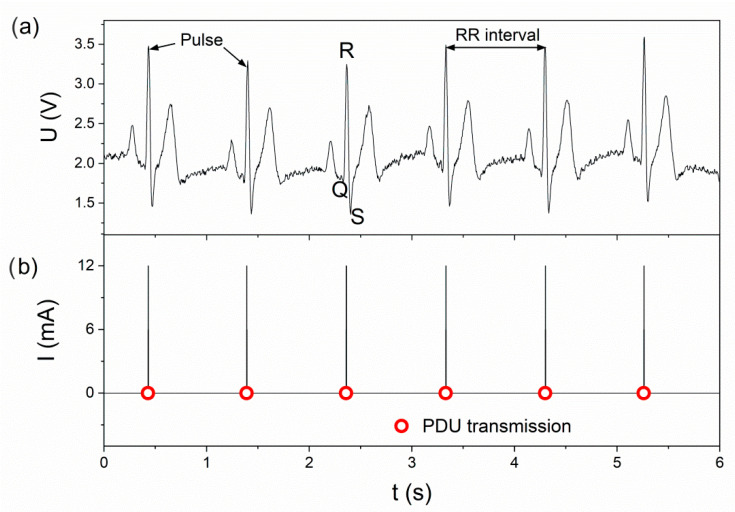
Visualization of pulse controlled episodic transmission (**a**) transmission control signal, (**b**) moments of detection and sending PDU.

Episodic transmission involves sending a teletransmission signal when a specific event occurs. This type of transmission can be used to monitor cyclic human activities, e.g., breathing or pulse. With regard to the BLE standard, the present paper assumes that when a specific event is detected, the PDU package will be sent in a connectionless broadcast mode. This transmission makes it possible to receive information from sensors without the need to create a dedicated radio link or a defined network structure. In addition, it allows for data reception from many sensors on many devices simultaneously (e.g., smartphones) [24]. Figure 10 shows exemplary visualization of pulse-related episodic transmission. Detection of the maximum QRS complex (Figure 10a) in the ECG signal sends a PDU package. The intervals between consecutive peaks (RR intervals [33]) are also transmission intervals. The package transmission moments are marked with circles. The transmission process is related to the impulse increase in current consumption by the BLE transmitter (Figure 10b) [25]. The process of PDU transmission itself carries information about the detection of individual (pulse) peaks, whereas the reception of the package on one or more devices enables us to reconstruct this information. In the case when it is sufficient to only monitor the transmission control episode, e.g., pulse—there is no need to decode the contents of the PDU package on the receiving device side, which simplifies the software structure of the event monitoring system. On the other hand, a broadcast package (Advertising PDU) in the BLE standard enables the transmission of 31 bytes of data [34], which can be used to send information about a different monitored value at the same time.

In this study, the transmission was controlled by the detected pulse (fast-varying component of the signal), whereas the PDU package additionally contained information on the slow-varying component, i.e., respiratory cycles. The results of the performed measurements are described in more detail in the next section. 

## 3. Results and Discussion

### 3.1. Analysis of Data from Inertial Sensors

**Figure 11:**
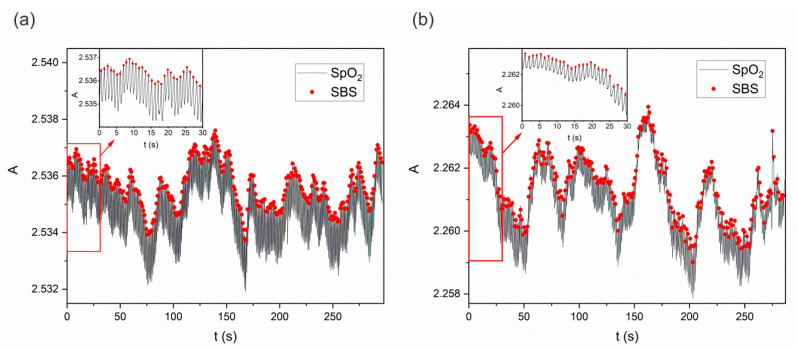
Results of the test performed in parallel with the use of the SpO_2_ sensor and the SBS inertial sensor, carried out by: (**a**) a woman (mean pulse 63 BPM), (**b**) a man (mean pulse 56 BPM). The signal amplitude is denoted as “A”.

Two healthy volunteers, a woman and a man, participated in the measurements. The measurements were carried out in accordance with the diagram presented in Figure 1. The inertial sensor (SBS—Figure 4) was placed on the anterior abdominal wall, whereas the SpO_2_ sensor (Figure 5) was placed under the index finger pad. The measurements with the use of both sensor types were performed in parallel for about 5 min. Figure 11 presents exemplary results from the measurements taken. In turn, the insets in Figure 11 represent the first 30 s of the measurement so as to show the shape of the signal read from the SpO_2_ sensor. Red dots mark moments when a pulse is detected by the inertial sensor (SBS). Since the measurements using the two methods (SpO_2_ and SBS) were performed in parallel, the timeline for both methods is the same. However, due to differences in the measurement method, only those values were selected from the IR signal that corresponded to the pulse detection time on the SBS (PDU reception time) in order to visualize the moment of pulse detection by the radio inertial sensor SBS against the IR signal (SpO_2_). It can be observed in Figure 11a,b that the moments of pulse detection by both methods differ slightly in time. However, it does not affect the determination of the mean pulse obtained by both methods. The episodic transmission process is connected in the discussed case with the detection of local signal maxima. In turn, the inaccuracies in the detection of individual peaks are related, among others, to signal distortion (artifacts) [35].

**Figure 12:**
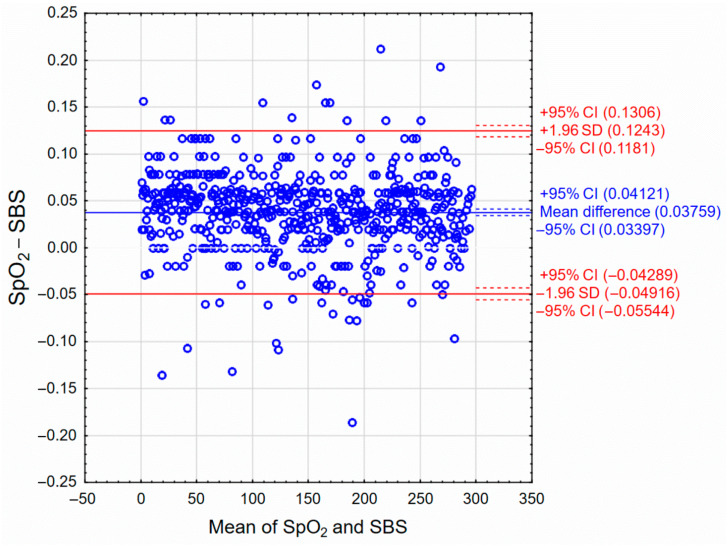
Bland-Altman graph evaluating the compatibility between measurements made using the SBS and the reference SpO_2_ sensor.

The Bland-Altman analysis [36] was used to assess the correspondence between the measurements made using the SBS and the reference SpO_2_ sensor, and its results are presented in Figure 12. The pulse detection times were compared, without separating the results by sex, as shown in Figure 11.

**Figure 13:**
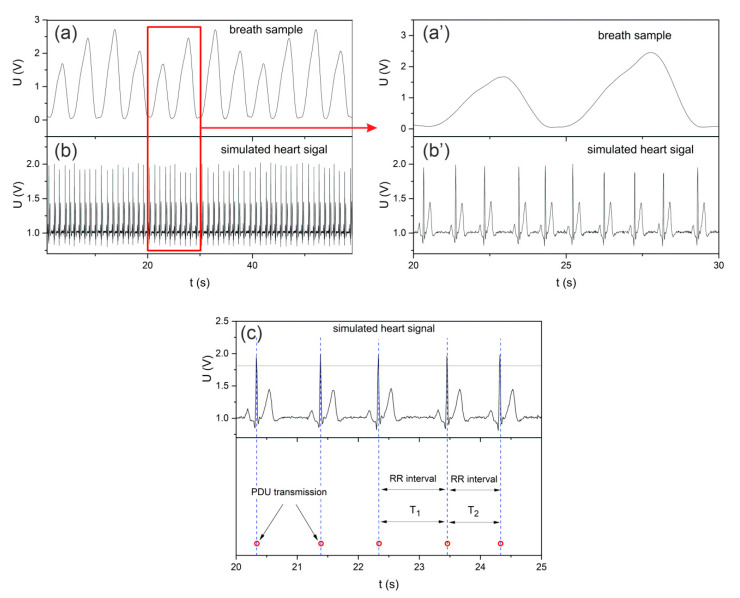
Exemplary scenario of using episodic broadcast transmission (**a**) respiratory signal BS stored in PDU, (**b**) control signal HS for BLE transmission, (**c**) description of parameters of the ECG signal controlling episodic broadcast transmission.

The time the PDU was obtained from the SBS is shifted relative to the time of pulse detection by the SpO_2_ sensor by an average of 0.038 s (referred to as “Mean difference” in Figure 12). The mean pulse from the measurements presented in Figure 11 was about 60 BPM, so the average time between successive peaks (cycle time) in the IR signal was 1 s. The difference of 0.038 s is, therefore, only about 4% of the average cycle time. Thus, the consistency of measurements between the two systems is very high. A positive difference value indicates a slight delay in pulse detection by the SBS compared to the SpO_2_ sensor. Figure 12 shows that only 4.7% of measurement exceeds the 95% compatibility interval (marked as “±1.96SD” in Figure 13). Therefore, these differences may result from something else than a measurement error. The remaining values are within the range (including Confidence Intervals) and there is no trend, so there is no relationship between the mean values from both sensors and the differences between them. 

### 3.2. Exemplary Scenario of Using Episodic Transmission

In order to present the functionality of BLE episodic broadcast transmission, the teletransmission system presented in Figure 3 was used. The HS and BS signals generated by the I/O Device were fed to the analogue inputs of the Transmitter BDK module. Figure 13a,b show the concurrently generated signals HS and BS. Measurements of the HS signal in the Transmitter BDK module were carried out every 0.01 s (100 Sa/s), whereas the measurements of the slow-varying BS signal were carried out every 0.1 s (10 Sa/s). The data from the BS respiratory signal measurements were saved in subsequent bytes of the Advertising Data of PDU section, whereas the data packets were transmitted when the maximum complex QRS peak of the ECG signal was detected. Thus, the detection of QRS peaks is related to the formation and sending of PDUs. For example, if the time (RR interval) between consecutive complex QRSs was 0.8 s, 8 bytes of data from the BS respiratory signal measurements were saved in the PDU. Since a deviation parameter (BPM Deviations—8%) was introduced, QRS peaks were generated every 1s (from approx. 0.9 to approx. 1.1 s). Thus, during the RR interval, it is possible to obtain 9 to 11 measurement results that will be saved in the PDU (9 to 11 bytes). Figure 13c shows the different times between the individual QRS cycles (RR intervals), which are also transmission intervals (T_1_, T_2_). In order to minimize the range of processed data, the detection of the QRS peak was above the defined threshold of 1.8 V, as indicated by the symbolic red line in Figure 13c. 

The performed tests also indicate that episodic BLE broadcast transmission is characterised by relatively low energy consumption. For the tests, a radio module containing the nRF52832 chip was used, which was configured in both connection and connectionless mode. The connection mode was configured with the standard settings of the radio module (based on the code examples provided by the manufacturer), where the connection interval ranges from 100 ms to 200 ms. In turn, the broadcast mode was configured to represent pulse-controlled transmission. The broadcast transmission interval was defined as 1 s, which corresponds to a mean pulse value of 60 BPM. The obtained results are presented in Table 1. During connectionless broadcast transmission using a single radio channel (analogous to the measurement stations in Figure 1a, Figure 2, and Figure 3), the radio module drew current (RMS curve) of 0.25 mA in pulses. On the other hand, the same module during connection transmission, with standard settings, consumed more than twice that current, i.e., 0.53 mA (by 112% more). Current consumption tests were also carried out with connectionless broadcast transmission with an interval of 1 s, where the BLE transmitter transmitted PDU on three channels, which allows for compatibility, among others, with smartphones. Then the current consumption during transmission was 0.41 mA. Connection transmission with standard transmitter settings requires approx. 29% higher current (0.53 mA). The BLE standard, whether in connection or connectionless broadcast mode, ensures efficient energy management. For example, according to the manufacturer’s documentation, a miniature and energy-saving Wi-Fi module, ESP07 with the ESP8266 chipset [37], consumes an average of 80 mA.

**Figure 14:**
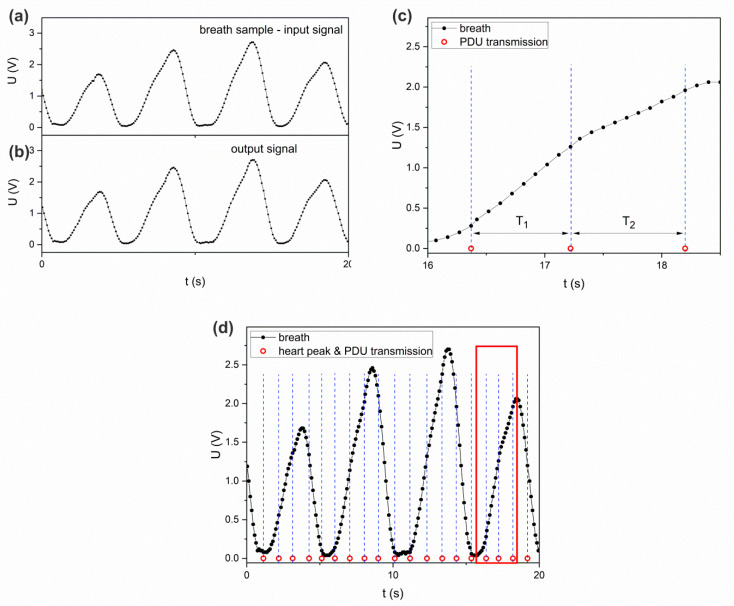
Curves representing the sent and received respiratory signals (**a**) radio-transmitted signal, (**b**) received signal, (**c**) reconstructed respiratory signal and marked PDU sending times, (**d**) visualization of a different number of measurement points for different transmission intervals.

Figure 14 shows curves representing the sent and received respiratory signals. The curve reproduced on the side of the BDK Receiver (Figure 14b) does not differ from the curve generated on the side of the BDK Transmitter (Figure 14a). The absence of differences between these curves indicates that the monitored signal (respiratory signal) is correctly mapped after episodic transmission on the receiver side. In turn, Figure 14c shows the reconstructed BS curve in the context of PDU transmission.

The times of sending the packets with the data of the respiratory function curve are marked with circles. The PDU is sent when the complex QRS peak is detected, and the receipt of the packet on the Receiver BDK side allows for the reconstruction of information about the pulse. As the packet transmission did not occur at equal time intervals (QRS peaks generated from BPM Deviations—8%), the reconstructed BS curve contains a different number of points at different transmission intervals (T_1_ and T_2_), as shown in Figure 14d. Each point of the reconstructed curve simultaneously corresponds to the data contained in a single PDU byte.

## 4. Conclusions

An important aspect of modern systems for monitoring body functions is the possibility of wireless data transmission. Currently, IoT solutions, including wearable sensors that work with mobile devices, e.g., smartphones, are being intensively developed. In such systems, it is important to reduce the energy demand, particularly on the side of battery-powered wearable sensors. Radio data transmission is one of the factors that significantly affects the energy demand of such solutions, therefore it is important to use energy efficient transmission methods and radio interfaces. One of the methods of reducing the energy demand of radio transmission is discrete transmission, used, for example, in the Bluetooth or Wi-Fi standard.

The paper presents examples of discrete BLE transmission controlled by biomedical signals. Transmission is managed with the use of pulse detection from sensor signals and simulated bioelectric signals. Discrete transmission controlled by pulse detection in the tested signals allows for effective monitoring of this episode (pulse) without the need to write data on the transmitter side and read the PDU content on the receiver side. In addition, the use of broadcast transmission enables us to monitor defined episodes on many devices at the same time, e.g., on smartphones, without the need to create a dedicated connection. The performed tests have shown high efficiency of pulse monitoring with the use of connectionless, BLE episodic broadcast transmission. In the proposed method of pulse monitoring, even noisy signals, e.g., from inertial sensors, can be used to control transmission. High efficiency of simultaneous monitoring of two parameters—pulse and breathing has also been demonstrated, where the pulse signal controlled the transmission, and the data on the slow-varying respiratory signal were saved in the PDU. The obtained results may provide a perspective for the use of episodic BLE transmission to monitor other body functions—e.g., gait cycles.

## 5. Patents

The described solution has obtained patent protection in Poland [38,39].

## Figures and Tables

**Table 1:** Summary of current consumption for various types of transmission.

Connection Transmission *	Connectionless Transmission Broadcast BLE		Wi-Fi ** Chipset ESP8266
	1 Channel	3 Channels	
0.53 mA	0.25 mA	0.41 mA	80 mA [37]

* Based on the code examples provided by the manufacturer. ** based on the manufacturer’s data.

## Data Availability

Not applicable.
